# In search of a ‘pan-European value set’; application for EQ-5D-3L

**DOI:** 10.1186/s12874-022-01830-3

**Published:** 2023-01-12

**Authors:** Ayesha Sajjad, Matthijs M. Versteegh, Irene Santi, Jan Busschbach, Judit Simon, Leona Hakkaart-van Roijen

**Affiliations:** 1 Erasmus School of Health Policy and Management, Erasmus University Rotterdam, Rotterdam, The Netherlands; 2 Institute for Medical Technology Assessment (iMTA), Erasmus University Rotterdam, Rotterdam, The Netherlands; 3 Section of Medical Psychology and Psychotherapy, Erasmus MC, Rotterdam, The Netherlands; 4 Department of Health Economics, Center for Public Health, Medical University of Vienna, Vienna, Austria; 5 Department of Psychiatry, University of Oxford, Oxford, UK

**Keywords:** Utilities, EQ-5D-3L, Value set, Time trade-off, Europe

## Abstract

**Objectives:**

Country-specific value sets for the EQ-5D are available which reflect preferences for health states elicited from the general population. This allows the transformation of responses on EQ-5D to health state utility values. Only twelve European countries possess country-specific value sets and no value set reflecting the preferences of Europe exists. We aim to estimate a ‘pan-European’ value set for the EQ-5D-3L, reflecting the preferences for health states of the European population that could help to evaluate health care from the perspective of the European decision-maker.

**Methods:**

We systematically assessed and compared the methodologies of available EQ-5D-3L time trade-off (TTO) value sets from twelve European countries: Denmark, France, Germany, Hungary, Italy, Netherlands, Poland, Portugal, Romania, Slovenia, Spain and UK. Using their published coefficients, a dataset with utility values for all 243 health states was simulated. Different modelling techniques and model specifications including interaction terms were tested. Model selection was based on goodness-of-fit criteria. We also explored results with application of population size weights.

**Results:**

Methodological, procedural and analytical characteristics of the included EQ-5D-3L valuation studies were quite comparable. An OLS based model was the preferred model to represent European preferences. Weighting with population size made little difference.

**Conclusions:**

EQ-5D-3L valuation studies were considered of sufficient comparability to form the basis for a new ‘pan-European’ value set. The method used allows for an easy update when new national value sets become available.

**Supplementary Information:**

The online version contains supplementary material available at 10.1186/s12874-022-01830-3.

## Plain english summary

Within Europe, country-specific value sets for EQ-5D are increasingly being utilized for economic evaluations. These national value sets are a helpful tool for international economic evaluations as more countries are initiating partnerships to enhance the access of patients to high quality and cost-effective treatments throughout Europe. In the absence of a national value set, investigators tend to use a value set from a neighbouring country as a proxy of the national-specific value set. The validity of the use of such proxies can be increased if existing value sets from different countries were combined to develop a ‘pan-European’ value set. This pan European value set combines utilities from valuation studies using Time Trade-Off (TTO) techniques from different European countries to provide means for standardizing multi-country economic evaluations. In our analysis, the OLS model comprised the pan-European value set. Potential use of this value set may help diminish country-specific differences in cost-effectiveness analyses from individual countries. This pan-European value set provides a feasible and pragmatic solution for international comparisons which could aid policy-making decisions across Europe.

## Introduction

In recent years, there has been an increase in the use of economic evaluations for the appraisal and strengthening of healthcare programs at national and multi-national levels [1]. Economic evaluations commonly use the Quality Adjusted Life-Years (QALY) as a measure of benefit to value health outcomes. Estimating QALYs requires the application of preference weights/utilities for health states in different populations, countries or regions. These preferences are measured with preference elicitation studies and subsequently captured in a so called ‘value set’. Currently the most used instrument to generate preference weights/utilities for these value sets is the EQ-5D [2, 3]. Many countries ideally have their own set of national values for the EQ-5D to ensure that resource allocation decisions based on economic evaluations reflect the preferences for health states of its own population. Value sets using the time trade-off (TTO) valuation technique for 3-level of the EQ-5D (EQ-5D-3L) now exist for twelve European countries [4–13]. For countries that do not yet have a national value set, it is common practice to use another country’s value set as a proxy. Currently, no definitive criteria exist to choose from for such a proxy value set.

An option for the use of proxy values of neighbouring or otherwise culturally related countries, might be the use of a pooled value set. Such a pooled value set would mitigate, to some extent, the variance due to methodological differences, but it will obviously also eliminate the possibility to account for differences in cultural values. After an extensive study, using several available international EQ-5D values sets, Roudijk et al. came to the conclusion that although differences between national values sets exits, these differences could not be explained by national cultural values [14]. The main difficulty the authors encountered was that the possible influence of national cultural values is nested with possible methodological variation between the national studies. However, this study included both 3L and 5L studies in their analyses, which made it hard to distinguish if variation between value sets was driven by national cultural values or by specific methodological differences at the level of national investigations.

Within the European context, a pooled value set may also inform health care policy and decision making at the European level, for example in determining the value of a vaccine in a multi-Member State procurement setting through health technology assessment (HTA) methods or for use with multi-national trials. Using one pooled European value set could be seen as a simple way to standardise health care evaluation with respect to Health Related Quality of Life (HRQoL). Indeed, organizations such as EUnetHTA and Beneluxa are promoting networks between European countries in order facilitate reliable, timely, transparent, and transferable information to contribute to HTA [1, 15]. A pooled European value set can support these efforts.

The aim of the present study is to derive a pooled value set from the published coefficients of TTO valuation studies of the EQ-5D-3L within Europe. We will refer to this pooled value set as a ‘pan-European’ value set. Our reasoning to use published coefficients to create such a value set is two-fold: methodological and practical. The methodological reasons include that when the raw national data is used, and international uniform in- and exclusion criteria are applied, the newly selected data is no longer similar to the one used in the original national valuation studies. That means consideration to ex- or included values at a national level are ignored, although they might be based on relevant local knowledge and preferences of the researchers involved. Moreover, model specifications that are considered relevant at national level are ignored. An alternative is to generate data from the published coefficients of the national studies, which are based on locally informed in- and exclusion criteria and reflect local considerations about the choices of the models. Practically, the developed methodology will allow for an easy update of the pan-European value set, when new national valuation studies become available. Even though, new valuation studies are focusing on generating health state preferences for the newly developed five-level version of the EQ-5D instrument, the 3-level version is also still in use, and EuroQol does not recommend one or the other. Therefore, we initialize to develop the pan-European value set for EQ-5D-3L; the resulting methodology can also accommodate an estimation of a pooled value set on the EQ-5D-5L [16].

## Methods

### EQ-5D-3L Time trade-off

Twelve European countries have developed TTO-based value sets of the EQ-5D-3L: Denmark, France, Germany, Hungary, Italy, Netherlands, Poland, Portugal, Romania, Slovenia, Spain and UK [4–13, 17, 18]. Ten of these studies used the Measurement and Valuation of Health (MVH) protocol from the UK tariff as a starting point, the valuation methodologies which were conducted to arrive at the value sets vary across studies [19]. The most recent Romanian and Hungarian studies used the EuroQol Valuation Technique (EQ-VT). In the Hungarian study, a 3L valuation was embedded in a valuation study of the 5L version of the EQ-5D, as a methodological add-on [11, 20, 21].

We systematically investigated the variation between studies by applying the checklist proposed by Attema et al. [22], which incorporates a list of choices to be made by researchers who wish to perform a TTO task. Such a checklist enables other researchers to align methodologies in order to enhance the comparability of health state values [22]. Methodological, procedural and analytical characteristics of the included EQ-5D-3L studies were extracted and compared (See Additional file: Tables 1–3).

### Statistical analyses

We simulated a so called ‘saturation data set’ by estimating the utilities for all 243 (3^5^) theoretical health states derived from the EQ-5D-3L using the coefficients from published TTO valuation studies from European countries: Denmark, France, Germany, Hungary, Italy, Netherlands, Poland, Portugal, Romania, Slovenia, Spain and UK (see Additional file: Table 4) [4–13, 17].

The saturated data set contains a unique value per health state for each country. As we estimated national values for twelve countries, we had 243 × 12 = 2916 data points forming a pooled data set of estimated TTO values. The dependent variable was the pooled utility, in which the best health state has an upper bound at 1, a negative lower bound for the worst imaginable health state and 0 being dead. The regressors were constructed as dummy variables to model the shift between the three levels of the EQ-5D-3L descriptive system within each of the five dimensions. Thus, two dummy variables were constructed for the Mobility dimension: one measuring the shift between level 1 and level 2 (MO2) and one measuring the shift between level 2 and level 3 (MO3). Similar dummy variables were constructed for other dimensions: Self-care (SC2, SC3); Usual Activities (UA2, UA3); Pain/Discomfort (PD2, PD3) and Anxiety/Depression (AD2, AD3).

We used different regression methods in order to select the functional form that best fitted the generated pooled data. We tested ordinary least squares (OLS) regression, generalized linear model (GLM) and finite mixture model (FMM).

### Ordinary least squares (OLS) 

OLS is the most commonly used linear least squares method for estimating the unknown parameters in a linear regression model.

### Generalized linear model (GLM)

GLM with Gamma distribution and a log-link was also tested. In this model, the disutility score (1-utility score) was entered as the dependent variable because positive values are required for this regression model.

### Finite mixture model (FMM)

**Fig. 1:**
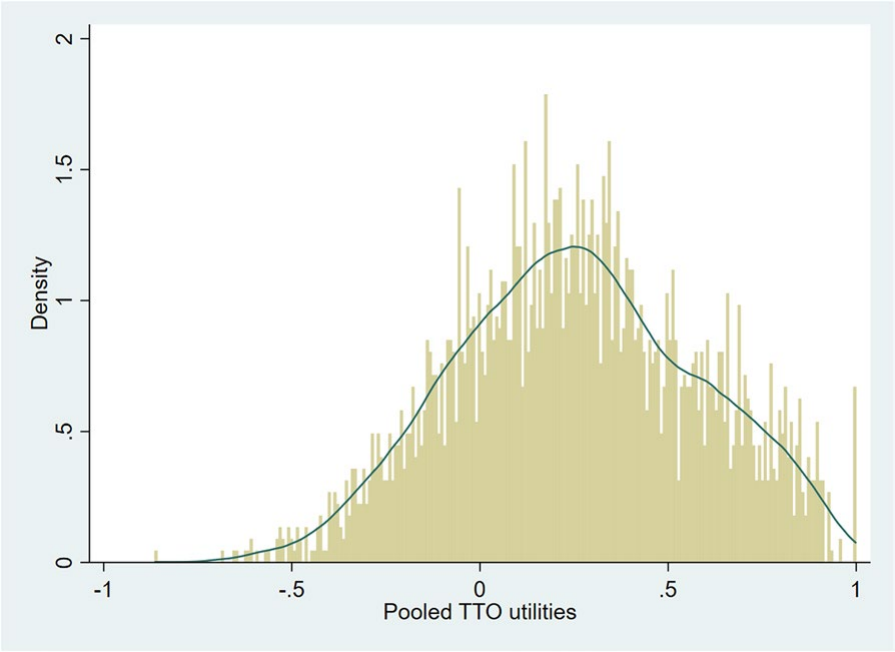
Kernel density plot of the saturated pooled utilities

The visual inspection of the distribution of the pooled EQ-5D-3L data revealed a mixture of at least two normal distributions, suggesting two distinct groups of utilities from the pooled countries. (Fig. 1). It is known that this pattern is a result of the EQ-5D-3L classification system, which generates differences between utilities with the same severity in respect of dimensions that are mainly observed at level 2 or 3. The weights commonly used to calculate the index exacerbate this grouping by placing a larger weight on level 3 dimension, generating a noticeable gap in index between the groups. Therefore, we also tested finite mixture models, which combines two or more probability density functions, making it capable of approximating any arbitrary distribution that may be relevant [23, 24]. Because of its flexibility, the FMMs are able to handle complex and multimodal distributions that often characterize HRQoL data, such as the EQ-5D [25]. A FMM, with two components of normal densities, was fit to the pooled utility data using the Stata™ command “fmm”.

The standard errors for all tested models were adjusted for clustering at the country level to account for differences in population sizes. Model selection for the best predictive model was based on goodness-of-fit criteria. Root mean square error (RMSE), R-squared (R^2^) and Akaike Information Criterion (AIC) were compared for the OLS model; RMSE, RMSE and pseudo R-squared (R.^2^) for the GLM. FMM were compared with other models through RMSE and AIC for model fit and estimation of prediction error. All analyses were performed using Stata17 (Stata Corp, College Station, TX) [26].

To illustrate the application of weights, we also estimated the regression models with weights for country population size, that were retrieved from Eurostat Census database using the year of publication of each country’s valuation set (see Additional file: Table 4). This was done for the weights to be broadly generalizable to the population sample in the respective country’s value set [27].

In a sensitivity analysis, we included testing for interaction terms identified in the included valuation studies: N3, D1, I2, and I3^2^ [6, 28]. They are created as dummy variables to account for overall aspects not indicated by the scores on the five dimensions of the EQ-5D-3L. N3 has a value of ‘1’ if any dimension is at level 3; 0 otherwise. D1 indicates the number of movements away from full health beyond the first. This refers to the number of dimensions that are level 2 or 3 and replaces the constant [28]. I2 indicates the number of dimensions at level 2 beyond the first. I3^2^ indicates the square of the number of dimensions at level 3 beyond the first.

## Results

The methodological, procedural and analytical characteristics of the included EQ-5D-3L studies were extracted and compared in accordance with the checklist proposed by Attema et al. (See Additional file: Tables 1–3) and are summarised below:

### Methodological characteristics

All studies except the Hungarian and Romanian value sets, were based on the British MVH protocol which was the first large-scale national study to elicit health state preferences from the EQ-5D-3L using the TTO method in 1997 [19]. The Hungarian and Romanian studies employed the composite TTO (cTTO) approach. The Hungarian study employed a 3L valuation as a methodological add-on to the EQ-VT protocol for EQ-5D-5L [11, 21]. In the MVH protocol, all studies estimated both “better than dead’ (BTD) and ‘worse than dead’ (WTD) states. All studies had 10 years of disease duration in the TTO task. Next to the similarities, there were also notable differences, or it was unclear if the protocol was followed strictly. For instance, the fixed TTO iteration procedure was abandoned in the Portuguese valuation study, and more freedom was given to the interviewers to employ a pragmatic ‘ping-pong’ approach in finding the maximum trade-off in the TTO exercise.

### Procedural characteristics

All studies used a representative sample of the national population for the TTO task. The sample size varied considerably between the countries, ranging from 0.002% of the population in the Dutch valuation study to 0.005% in the UK study. The Dutch study directly valued the least number of observed health states: 17 and Denmark the most: 46. All studies conducted face-to-face interviews for the TTO tasks. Denmark, The Netherlands and France performed the TTO valuation on the computer. The Hungarian study used the composite time trade-off (cTTO) valuation on the computer. The cTTO approach combines conventional 10-year TTO to elicit values for BTD health states and a lead-time TTO for WTD health states [29]. All studies administered a Visual Analogue Scale and/or a ranking task before the main TTO exercise, as a warm-up task to familiarize the respondents with the TTO exercise. Detailed differences in the sampling and TTO administration of the included valuation studies are provided in Additional file: Table 2.

### Analytical characteristics

Additional file: Table 3 presents the analytical properties of included valuation studies. The criteria defined to exclude participants in the TTO task differed in each country’s valuation study. Respondents with incomplete/missing TTO data and extreme values on the TTO task were excluded from the analyses. Data on all health states given the same value and those states which were valued worse than dead were also excluded. Spain, Poland and Portugal excluded the respondents who presented with “serious” logical inconsistencies. These logical inconsistencies were defined as when the respondent values a logically better state worse than the worse state. A logical inconsistency was called “serious” if the difference in valuation was greater than 0.5. All studies used transformations for health states valued worse than death, so that they were bounded by a maximum negative value of -1. The mean negative values are therefore between -1 and 0. In the cTTO approach used in the Hungarian and Romanian studies, WTD values tended to be higher than the traditional TTO (tTTO) values [30].

### A pan-European value set for EQ-5D-3L

**Fig. 2:**
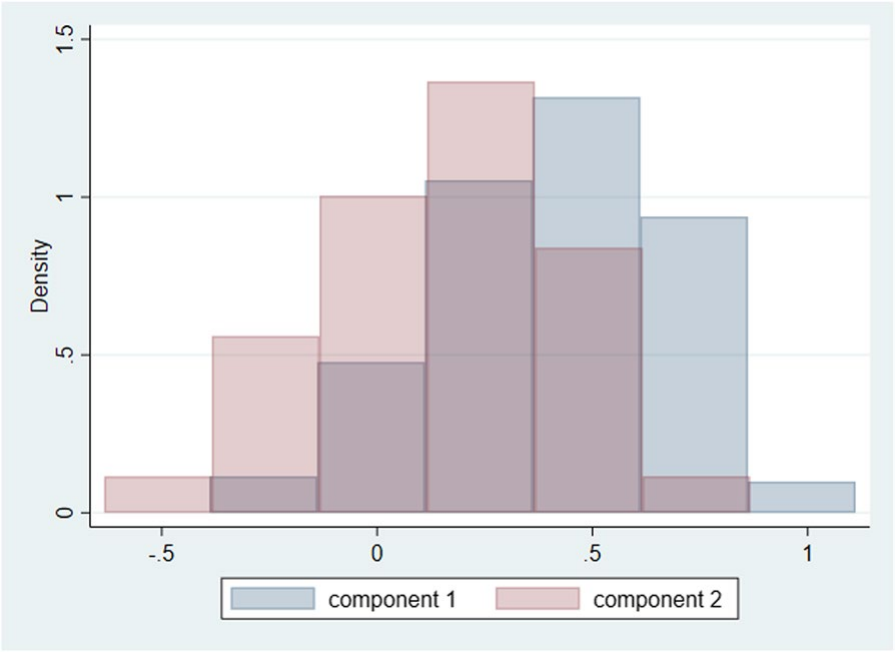
Predicted utilities for 2 components – FMM model

The range of utility values produced by the twelve European valuation studies was from -0.865 to 1.000 (Fig. 1). Table 1 presents the goodness of fit statistics of different functional forms tested on the pooled saturated data synthesized from the coefficients of existing EQ-5D-3L value sets. Out of the three models tested, FMM had the lowest value for AIC (-3401.044) reflecting the best fit, but the largest value for RMSE (0.213) which reflects that among all models, the predicted values from the FMM model were furthest from the observed values. Furthermore, application of population weights resulted in lack of convergence. The OLS regression had the second-best goodness of fit (based on RMSE, R-squared and AIC values; Table 1) and was therefore the preferred regression technique. The residuals-versus-fitted values to assess homoscedasticity were plotted and showed no visible pattern (see Additional file: Fig. 1). Additionally, the BreuschePagan/ CookeWeisberg test for heteroskedasticity of the error terms was conducted and resulted to be non-significant (*p* = 0.8452), indicating that the error variances are all equal and that the homoskedasticity assumption of OLS holds. The values from this model best represent the preferences of the European population and thus comprise a pan-European value set. Table 2 presents the unweighted regression coefficients from the selected model which is derived from the pooled estimates with all selected interaction terms from the OLS regression analyses. Table 2 also includes and population size weighted regression coefficients from the OLS regression analyses, together with the unweighted regression coefficients from the component 2 of the FMM model. As from the two-component FMM model, the latent class probabilities show higher prediction for component 2 (60%, Fig. 2), therefore the coefficients of component 2 are also presented in Table 2 only for comparison. See Additional file: Table 5 for the coefficients of both components 1 and 2. The results of the sensitivity analysis that includes inclusion of interaction terms to the OLS model with and without application of population weights is also presented in Additional file: Table 6.

**Table 1:** Goodness of fit statistics of the tested regression techniques and OLS models including interaction terms

Goodness of fit statistics of the tested regression techniques				
	**RMSE**	**Pseudo-R**^**2**^	**R**^**2**^	**AIC**
OLS	0.151	-	0.785	-2739.665
GLM with gamma log link	0.202	0.693	-	-
FMM	0.213	-	-	-3401.044

*RMSE* Root mean square error, *R*^*2*^ R-squared, *AIC* Akaike information criterion, *OLS* Ordinary least squares, *GLM* Generalized linear model, *FMM* Finite mixture model

**Table 2:** Unweighted FMM, unweighted and weighted OLS (with application of population weights) to estimate the ‘pan-European’ value set for EQ-5D-3L

	**OLS model (unweighted)**			**OLS model (applied population weights)**			**FMM model (unweighted)**		
	**β**	**Std. Err**	**P value**	**β**	**Std. Err**	**P value**	**β**	**Std. Err**	**P value**
**MO2**	-0.074	0.013	0.000	-0.085	0.017	0.001	-0.090	0.020	0.000
**MO3**	-0.422	0.033	0.000	-0.391	0.023	0.000	-0.428	0.045	0.000
**SC2**	-0.087	0.014	0.000	-0.101	0.024	0.001	-0.105	0.023	0.000
**SC3**	-0.258	0.019	0.000	-0.261	0.017	0.000	-0.262	0.020	0.000
**UA2**	-0.051	0.011	0.001	-0.052	0.026	**0.067**	-0.063	0.022	0.003
**UA3**	-0.178	0.017	0.000	-0.149	0.030	0.000	-0.186	0.022	0.000
**PD2**	-0.083	0.007	0.000	-0.089	0.011	0.000	-0.084	0.011	0.000
**PD3**	-0.348	0.022	0.000	-0.353	0.030	0.000	-0.298	0.037	0.000
**AD2**	-0.056	0.011	0.000	-0.044	0.017	0.028	-0.048	0.022	0.028
**AD3**	-0.228	0.023	0.000	-0.189	0.023	0.000	-0.202	0.038	0.000
**Constant**	0.852	0.029	0.000	0.862	0.027	0.000	0.743	0.079	0.000

^*^P > 0.05; MO2 1 if mobility is level 2; 0 otherwise All models

MO3 1 if mobility is level 3; 0 otherwise All models

SC2 1 if self-care is level 2; 0 otherwise All models

SC3 1 if self-care is level 3; 0 otherwise All models

UA2 1 if usual activities is level 2; 0 otherwise All models

UA3 1 if usual activities is level 3; 0 otherwise All models

PD2 1 if pain/discomfort is level 2; 0 otherwise All models

PD3 1 if pain/discomfort is level 3; 0 otherwise All models

AD2 1 if anxiety/depression is level 2; 0 otherwise All models

AD3 1 if anxiety/depression is level 3; 0 otherwise All models

The regression coefficients of UA2 (moderate problems in usual activities) in the weighted model did not reach statistical significance with application of population weights (*p* > 0.05). This suggests that the weighting complicated the model. Notably, differences between the weighted and non-weighted results were limited.

## Discussion

This study compared methodological, procedural and analytical characteristics of the twelve EQ-5D-3L TTO valuation studies. Differences existed in sample size, the number of health states valued and exclusion criteria. All except the Hungarian and Romanian valuation studies were based on the MVH protocol. All studies used the additive 10-parameter model, which represents levels 2 and 3 for each dimension except for the Slovenian study which used a constrained 6-parameter model approach that assumed the relative severity of level 2, “moderate problems”, being similar across dimensions. This method was used in the Slovenian value set due to concerns about the relatively small sample size and limited number of valued health states. Furthermore, in the Polish, Dutch and Italian studies, the translations chosen for describing the levels of severity in health states may render differences in comparison with other value sets. For instance, in the Polish value set; mobility level 3 “confined to bed,” implied being bedridden, therefore, the Polish values may be lower for health states that included level 3 of mobility. However, these differences in valuation techniques and methodologies did not hinder us from pooling the utility values that each country is using for their respective HTA. Based on the published coefficients, we were able to simulate a dataset on which we could estimate the ‘pan-European’ value set. The resulting coefficients can be applied when national values are absent. The pan-European value set would also be an optimum choice when decisions need to consider a European perspective, for instance, for reimbursement decisions at the European level. This contributes towards cross-country harmonization of outcome measures for economic evaluations [31].

As this study aims to provide a means for standardizing multi-country evaluations by combining valuation tariffs from different countries in a particular region (e.g. Europe), one obvious factor to consider is the varying population size of different European countries included in this analysis. In order to account for differences in population size, we applied population size weights, adjusted for clustering at the country level. We found that including these weights for population size complicated the modelling. This may be related to the coefficients of the German value set, which is known to have the highest values, and is weighted with the highest population size [9, 32]. This weighting therefore introduces considerable variance, while it is unclear whether these high values truly represent higher values of the German population, or that the high values are an artefact of the sampling technique employed in the study. Indeed, when catering values for the new EQ-5D-5L, a decade later, the German values converge with other values from European value sets which suggests that the first attempt with the EQ-5D-3L had methodological issues [33].

Given the reasoning above, the application of population size weights in our study should be considered as an illustrative example. When it comes to weighting value sets of different countries, other factors such as socio-demographic, societal, religious, economic and linguistic factors can be included as weights as they may further explain inter-country differences [34]. A flexible modelling technique which can easily incorporate these weights would be helpful to guide our choice of the OLS model to predict the pan-European value set for EQ-5D-3L. Nevertheless, given the small changes in the coefficients, as found in this study, it needs to be investigated whether the incorporation of weights for background variables increases the validity, or rather complicates interpretations. Therefore, application of weights in the analyses and their interpretation may need to be treated with caution.

We used EQ-5D-3L as an illustrative example in the exercise to estimate a pan European value set because of its widespread application in Europe. The same methodology can be applied for the new five level 5-level version of the EQ-5D, or with any other utility questionnaire that uses regression analytical techniques to estimate a value set.

Various previous studies have compared different EQ-5D valuations in an attempt to unify EQ-5D data and generate preference weights for regional general populations. Greiner et al. were one of the first to derive European weights using the EQ-5D – Visual Analogue Scale (VAS) data from 11 European countries [35]. Time trade-off data is preferred over VAS data as this valuation method asks respondents to make a trade-off between the attributable time and HRQoL, much in the same way as a QALY can be interpreted. Olsen et al. also compared time trade-off valuations in four Western countries and three non-Western countries. They concluded that between the four European countries, there is less variance than between value sets of Western and non-Western value sets [36]. Another study compared three EQ-5D valuations in Central and Eastern European countries and further estimated a population norm for this region [37]. These studies thus suggest that a pooled value set depicting averaged European health state values may indeed be a feasible and sensible way forward in health economics research.

Some strengths and limitations merit consideration: despite of the differences among the included valuation studies, we present a flexible approach using published coefficients, which can accommodate more value sets as soon as they become available. This is a pragmatic approach that suggests that coefficients from existing published valuation studies could be combined to generate health state preferences for any specific region, being this Europe or any other geographical area or a sub-set of countries.

One can argue that a starting point for deriving a pooled value set should be the raw data of each country’s national valuation study [14]. However, the major disadvantage with this approach is that data collection for this study depended on the willingness of authors and institutes to share the data. Moreover, data sharing could be limited by constrains enforced by the informed consent, as the data is used for different purposes as described in the informed consent and data is transferred to others than the original research team, which may initiate privacy infringing.

In this study we applied and compared OLS regression, gamma regression, and FMM to best fit the pooled saturated data. We present the pan-European value set using the OLS which was the most pragmatic choice according to goodness of fit, prediction error and model convergence. Even though, the FMM model performs slightly better than the OLS model based on the penalized likelihood criteria (AIC) the model did not achieve convergence after the application of population weights. Therefore, further research into advanced analytic techniques is needed to test various model specifications using the FMM which are beyond the scope of the current paper. Future research to test different hypothesis, for instance, that the probability of belonging to a particular group (class) could also be consequently tested.

We included a sensitivity analysis with addition of the identified interaction terms from the existing valuation studies to the OLS model. Various interaction terms are used in some of the older EQ-5D-3L valuation studies such as N3, I2, I3^2^, D1 However, such interaction terms are not recommended to be included in models in recent valuation studies as they could increase the misprediction errors [38]. Furthermore the use of D1 interaction term has been heavily criticized as it may complicate the model [39].

The UK is no longer a part of the European Union (EU). This also entails that the UK is no longer a part of the regulations regarding therapeutic products, interventions, and evaluations of their effectiveness within the European economic area. Therefore, taking Brexit into consideration, we re-ran the OLS model as a scenario analysis with exclusion of the UK value set. The resulting pan-EU value set can be used for economic evaluations of drugs within the EU context (see Additional file: Table 7).

A limitation of this study is that the samples included in each valuation set were not entirely representative of the general population of the corresponding country [35]. Since some of the value sets are quite old, it is also questionable whether these value sets are still representative of the values of the general population, as population structure in the respective countries have changed over the years. Furthermore, societal differences such as educational status, culture, norms, wealth and on the other hand methodological differences such as elicitation methods, modelling, and quality of data may have influenced the health state valuations at individual country level. We identified that each valuation study had its unique characteristics, its own methodological framework and reasons for inclusions/exclusions. We also recognize that the quality of some value sets may be questionable. For instance, there are inconsistencies within the Portuguese value set where the value of health state 33,331 (- 0.536) is lower than the value of 33,333 ( -0.496). One approach to account for such differences would be to derive a quality score and further adjusting the analyses for it. However, we argue against this approach because the identified differences between studies might be more often properties which solely represent the thorough understanding of the respective country’s preferences rather than differences in quality.

## Conclusions

Our results suggest that when using the estimated combined health state values of the European countries, the OLS best represented the overall model and preferences of the researchers within the respected countries. Many societal and demographic factors may drive health preference differences between European countries, but difference may also be driven by artefact, difference in sampling or another methodological characteristic. A ‘pan-European’ value set may help diminish such country-specific differences in cost-effectiveness analyses from individual countries. This may provide a feasible and pragmatic solution for HTA bodies to allow for international comparisons thus aiding policy-making decisions across Europe and/or the European Economic Area/EU. Future studies could explore different advanced analytic techniques for better model fitting with adjustments for relevant societal and demographic variables. Lastly, identification of factors that can lead to geographical subgrouping of these analyses to develop ‘supra-national’ would be extremely useful.

## Supplementary Information

**Additional file 1.**

## Data Availability

This is a secondary analysis; no raw data was used.

## Abbreviations

TTO: Time Trade-off
QALY: Quality Adjusted Life-Years
HTA: Health Technology Assessment
HRQoL: Health Related Quality of Life.
MVH: Measurement and Valuation of Health
EQ-VT: EuroQol Valuation Technique
MO: Mobility
SC: Self-care
UA: Usual Activities
PD: Pain/Discomfort
AD: Anxiety/Depression
OLS: Ordinary Least Squares
GLM: Generalized Linear Model
FMM: Finite Mixture Model
RMSE: Root mean square error
R^2^: R-squared
AIC: Akaike Information Criterion
cTTO: Composite Time Trade-off
VAS: Visual Analogue Scale
