# Supplementary material for: In search of a ‘pan-European value set’; application for EQ-5D-3L
Source: BMC Med Res Methodol. 2023 Jan 12;23:13. doi: 10.1186/s12874-022-01830-3 (PMC9835298; doi:10.1186/s12874-022-01830-3)
Supplement: Supplementary file 1 — Additional file 1. [file 12874_2022_1830_MOESM1_ESM.docx]

**ADDITIONAL INFORMATION**

**In search of a ‘pan-European value set’; application for EQ-5D-3L**

Ayesha Sajjad MD, MPhil, PhD^1*^, Matthijs M. Versteegh PhD^2^, Irene Santi PhD^2^, Jan Busschbach PhD^3^, Judit Simon MD, PhD^4,5*^, Leona Hakkaart-van Roijen PhD^1,2*^

^1^Erasmus School of Health Policy and Management, Erasmus University Rotterdam, Rotterdam, The Netherlands; ^2^institute for Medical Technology Assessment (iMTA), Erasmus University Rotterdam, Rotterdam, The Netherlands; ^3^Section of Medical Psychology and Psychotherapy, Erasmus MC, Rotterdam, The Netherlands; ^4^Department of Health Economics, Center for Public Health, Medical University of Vienna, Vienna, Austria; ^5^Department of Psychiatry, University of Oxford, Oxford, UK

**Correspondence:** Dr. Ayesha Sajjad, Erasmus School of Health Policy and Management, Erasmus University Rotterdam, Rotterdam, The Netherlands. P.O. Box 1738, 3000 DR Rotterdam, The Netherlands. Email: [sajjad@eshpm.eur.nl](mailto:sajjad@eshpm.eur.nl); ayesha_sajjad@hotmail.com; Tel: +31 10 40 89656

**TABLES**

**Table 1** Methodological aspects of EQ-5D-3L valuations studies in ten European countries

|  | **Denmark** | **France** | **Germany** | **Hungary** | **Italy** | **Netherlands** | **Poland** | **Portugal** | **Romania** | **Slovenia** | **Spain** | **UK** |
| --- | --- | --- | --- | --- | --- | --- | --- | --- | --- | --- | --- | --- |
| **Value range spanned** | Both BTD and WTD | Both BTD and WTD | Both BTD and WTD | Both BTD and WTD | Both BTD and WTD | Both BTD and WTD | Both BTD and WTD | Both BTD and WTD | Both BTD and WTD | Both BTD and WTD | Both BTD and WTD | Both BTD and WTD |
| **Method used for valuation of WTD states** | MVH protocol | MVH protocol | MVH protocol | 3L methodological add-on to the EQ-VT protocol for EQ-5D-5L | MVH protocol | MVH protocol | Modified MVH protocol | MVH protocol | EQ-VT protocol | MVH protocol | MVH protocol | MVH protocol |
| **Time frame (Disease duration)** | 10 years | 10 years | 10 years | 10 years | 10 years | 10 years | 10 years | 10 years | 10 years | 10 years | 10 years | 10 years |
| **Smallest tradable unit listed** | No | Yes | Yes | Yes | Yes | Yes | Yes | Yes | Yes | Yes | Yes | Yes |
| **Iteration procedure** | Not specified | MVH fixed sequence | Not specified | cTTO iterative procedure: *x* is varied to identify the respondent’s point of indifference where the health state value is given by *x*/10 | MVH fixed sequence | MVH fixed sequence (outward titration) | Not specified | Ping-pong procedure | cTTO iterative procedure: x is varied to identify the respondent’s point of indifference where the health state value is given by x/10. | MVH fixed sequence | Not specified | MVH fixed sequence |
| **Response scale** | Years lived in full health | Years lived in full health | Years lived in full health | Years lived in full health** | Years lived in full health | Years lived in full health | Months and weeks lived in full health* | Years lived in full health | Years lived in full health | Years lived in full health | Years lived in full health | Years lived in full health |

***** when no valuation changes were noted for 9 years on 1 side of the time board. Results of the TTO exercise were read out from the scale in the protocol book with an accuracy of 0.25 of a year.
** The cTTO approach combines conventional 10-year TTO for health states considered better than dead (BTD) and lead-time TTO (ie, 10 years in full heath followed by 10 years in a less than healthy EQ-5D state) for health states considered worse than dead (WTD). cTTO offers respondents the conventional task comprising a series of adaptive choices between x years in full health and 10 years in the disease state.

**Table 2** Procedural aspects of EQ-5D-3L valuations studies in ten European countries

|  | **Denmark** | **France** | **Germany** | **Hungary** | **Italy** | **Netherlands** | **Poland** | **Portugal** | **Romania** | **Slovenia** | **Spain** | **UK** |
| --- | --- | --- | --- | --- | --- | --- | --- | --- | --- | --- | --- | --- |
| **Mode of administration?** | Face-to-face interviews – computer assisted | Face-to-face interviews | Face-to-face interviews | Face-to-face interviews – computer assisted | Face-to-face interviews – computer assisted | Face-to-face interviews | Face-to-face interviews | Face-to-face interviews | Face-to-face, computer-assisted, | Face-to-face interviews | Face-to-face interviews | Face-to-face interviews |
| **Visual aids used** | TTO board and health state cards – computer assisted* | computer assisted | TTO board and health state cards | Composite time trade-off (cTTO) valuation on the computer | TTO board and health state cards | TTO valuation was done on the computer | TTO board and health state cards | TTO board and health state cards | Composite time trade-off (cTTO) valuation on the computer | TTO board and health state cards | TTO board and health state cards | TTO board and health state cards |
| **Context effects considered** | Warm-up task: other utility valuations (ranking task, VAS) | Warm-up task: other utility valuations (ranking task, VAS) | Warm-up task: other utility valuations (ranking task, VAS) | Two wheelchair examples | Warm-up task: other utility valuations (ranking task, VAS) | Warm-up task: other utility valuations (ranking task, VAS) | Warm-up task: TTO  Ordering of states | Warm-up task: other utility valuations (ranking task, VAS) | cTTO warm up task – wheelchair examples | Warm-up task: Ranking task, VAS | Warm-up task: other utility valuations (ranking task, VAS) | Warm-up task: VAS |
| **Sampling frame** | General population | General population | General population | General population | General population | General population | General population | General population | General population | General population | General population | General population |
| **Observed health state values** | Indirect valuations | Indirect valuations | Indirect valuations | Indirect valuations | Indirect valuations | Indirect valuations | Indirect valuations | Indirect valuations | Indirect valuations | Indirect valuations | Indirect valuations | Indirect valuations |
| **Modelling approach** | Statistical inference | Statistical inference | Statistical inference | Statistical inference | Statistical inference | Statistical inference | Statistical inference | Statistical inference | Statistical inference | Statistical inference | Statistical inference | Statistical inference |
| **No. of included respondents** | N=1,322 | N=443 | N=339 | N=1000 | N=439 | N=298 | N=305 | N=417 | N=1556 | N=225 | N=975 | N=2,997 |
| **Number of health states to be valued** | 16 per respondent; 46 overall observed | 17 per respondent; 24 overall observed | 15 per respondent; 43 overall observed | 3 per respondent; 30 overall observed | 16 per respondent;  24 overall observed | 17 per respondent;  17 overall observed | 23 per respondent; 44 overall observed | 7 per respondent; 24 overall observed | 30 overall observed | 15 per respondent; 23 overall observed | 13 per respondent; 43 overall observed | 13 per respondent; 42 overall observed) |
| **Lowest health state in the valuation task** | PITS 33333 | PITS 33333 | PITS 33333 | PITS 33333 | PITS 33333 | PITS 33333 | PITS 33333 | PITS 33333 | PITS 333333 | PITS 33333 | PITS 33333 | PITS 33333 |
| **Model fit criteria** | R^2^ | MAE, AIC, R^2^ | R^2^ | MAE | MAE, AIC, BIC, R^2^ | MAE | MAE, R^2^ | MAE, R^2^ | AIC, BIC | MAE, MSE Lin’s Concordance Correlation Coefficient. | R^2^ | R^2^ |

TTO Time trade-off; VAS Visual analogue scale; R^2^ R-squared; MAE Mean absolute error; AIC Akaike information criterion; BIC Bayesian information criterion; MSE Mean square error

**Table 3** Analytical aspects of EQ-5D-3L valuations studies in ten European countries

|  | **Denmark** | **France** | **Germany** | **Hungary** | **Italy** | **Netherlands** | **Poland** | **Portugal** | **Romania** | **Slovenia** | **Spain** | **UK** |
| --- | --- | --- | --- | --- | --- | --- | --- | --- | --- | --- | --- | --- |
| **Exclusion criteria** | Not specified | Completely missing TTO data; Only 1 or 2 states valued; All states given the same value; All states valued as worse than dead. | Extreme values of the available TTO-data were determined and excluded | No exclusions were made on the basis of data quality, inconsistent responses or non-trading. | Completely missing TTO data; only one or two states assessed; all states reporting the same value; and all states valued as worse than  dead | Completely missing TTO data; All states given the same value | Fewer than three  states valued; all states valued worse than dead; all states valued  the same, and ‘logical and serious logical inconsistencies.’; Extreme values, defined as values more than 2 SD from the mean | Responses that were not considered to be rational trade-offs;  TTO data missing for all states; only one or two states valued; all states were given the same TTO value; and all states were valued worse than death; ‘logical and serious logical inconsistencies’ in the data | Interviews of suspect quality; Interviews performed by interviewers having more than 40% of the interviews performed flagged as interviews of suspect quality; Interviews performed by interviewers not performing enough interviews (<20)_ to achieve a harmonized learning effect between interviewers; Interviews for which the interviewer had not shown the WTD example in the training part of the survey; Participants with a positive slope on the regression between their values and the misery index of the health states assessed for participants who gave the same value to all health states or did not trade time (non-traders). | Not specified | Inconsistencies in health state valuations | Incomplete TTO data; |
| **Definition of best possible health** | Perfect health | Perfect health | Perfect health | Perfect health | Perfect health | Perfect health | Perfect health | Perfect health | Perfect health | Perfect health | Perfect health | Perfect health |
| **Analysis of WTD values*** | Transformation:  (*x*/10)-1 | Transformation:  -x/(10 - x) | Transformation:  (*x*/10)-1 | Transformation:  (*x* - 10)/10 | Transformation:  -*x*/10 | Transformation:  -*x*/(10 - *x*) | Transformation:  (*x*/10)-1 | Transformation:  (*x*/10)-1 | Transformation:  (*x* - 10)/10 | Transformation:  -x/(10-x) | 2 Transformations:  -*x*/(10 - *x*);  (*x*/10) – 1 | Transformation:  (*x*/10)-1 |

*Transformations: where *x* represents the number of years spent in full health (11111)

| **Regression Coefficients EQ-5D-3L** | | | | | | | | | | | | | |
| --- | --- | --- | --- | --- | --- | --- | --- | --- | --- | --- | --- | --- | --- |
|  | **Denmark** | **France** | **Germany** | **Hungary** | **Italy** | **Netherlands** | **Poland** | **Portugal** | **Romania** | **Slovenia** | **Spain** | **UK** |  |
| **Year of publication** | 2009 | 2011 | 2004 | 2020 | 2013 | 2006 | 2010 | 2013 | 2021 | 2020 | 2001 | 1997 |  |
| **Average population** | 5,523,095 | 65,127,852 | 82,516,260 | 9,769,526 | 60,233,948 | 16,346,101 | 38,042,794 | 10,457,295 | 19,186,201 | 22,080,908 | 40,850,412 | 58,316,954 |  |
| **Modelling technique** | Random Effects Regression | Random Effects Regression | Random Effects Regression | Tobit Regression | Random Effects Regression | Random effects regression | Random Effects Regression | Random Effects Regression | Interval Regression model | 6-parameter constrained regression model | Random Effects Regression | Generalized  least-squares regression |  |
| **constant** | 0.1137 | 0.187 | 0.001 | 0.980 | - | 0.071 | 0.049 | - | 0.032 | - | 0.024 | 0.081 |  |
| **MO2** | 0.0532 | 0.154 | 0.099 | 0.022 | 0.076 | 0.036 | 0.052 | 0.305 | 0.038 | 00.943 | 0.106 | 0.069 |  |
| **MO3** | 0.411 | 0.373 | 0.329 | 0.648 | 0.518 | 0.161 | 0.331 | 0.890 | 0.394 |  | 0.430 | 0.314 |  |
| **SC2** | 0.0629 | 0.210 | 0.087 | 0.051 | 0.100 | 0.082 | 0.054 | 0.354 | 0.040 | 00.243 | 0.134 | 0.104 |  |
| **SC3** | 0.192 | 0.325 | 0.174 | 0.355 | 0.289 | 0.152 | 0.235 | 0.796 | 0.206 |  | 0.309 | 0.214 |  |
| **UA2** | 0.0478 | 0.156 | - | 0.025 | 0.085 | 0.032 | 0.046 | 0.298 | 0.044 | 00.202 | 0.071 | 0.036 |  |
| **UA3** | 0.144 | 0.188 | - | 0.246 | 0.198 | 0.057 | 0.212 | 0.759 | 0.189 |  | 0.195 | 0.094 |  |
| **PD2** | 0.0623 | 0.110 | 0.112 | 0.080 | 0.098 | 0.086 | 0.057 | 0.306 | 0.072 | 00.448 | 0.089 | 0.123 |  |
| **PD3** | 0.396 | 0.264 | 0.315 | 0.338 | 0.334 | 0.329 | 0.489 | 0.703 | 0.371 |  | 0.261 | 0.386 |  |
| **AD2** | 0.0682 | 0.088 | - | 0.078 | 0.095 | 0.124 | 0.026 | 0.233 | 0.054 | 00.239 | 0.062 | 0.071 |  |
| **AD3** | 0.367 | 0.204 | 0.065 | 0.258 | 0.213 | 0.325 | 0.207 | 0.632 | 0.206 |  | 0.144 | 0.236 |  |
| **N3** | - | -0.169 | 0.323 | - | - | 0.234 | - | - |  |  | 0.291 | 0.269 |  |
| **D1** | - | - | - | - | -0.043 | 0.38 | - | -0.437 |  |  | 0.60 | 0.46 |  |
| **I2** | - | - | - | - | - | - | - | 0.240 |  |  | - | - |  |
| **I3^2^** | - | - | - | - | - | - | - | 0.034 |  |  | - | - |  |
| **R^2^** | - | - | - | - | 0.390 | 0.38 | 0.452 | 0.37 |  |  | 0.60 | 0.46 |  |

**Table 4** Regression coefficients from published EQ-5D-3L valuations studies in European countries

MO3 1 if mobility is level 3; 0 otherwise All models; SC2 1 if self-care is level 2; 0 otherwise All models; SC3 1 if self-care is level 3; 0 otherwise All models;UA2 1 if usual activities is level 2; 0 otherwise All models; UA3 1 if usual activities is level 3; 0 otherwise All models; PD2 1 if pain/discomfort is level 2; 0 otherwise All models; PD3 1 if pain/discomfort is level 3; 0 otherwise All models; AD2 1 if anxiety/depression is level 2; 0 otherwise All models; AD3 1 if anxiety/depression is level 3; 0 otherwise All models; N3 1 if any dimension is level 3; 0 otherwise; D1 Number of movements away from full health beyond the first (and replaces the constant); I2 Number of dimensions at level 2 beyond the first; I3^2^ The square of the I3: Number of dimensions at level 3 beyond the first.

| **Class 1** | **β** | **Std. Err** | **P value** |
| --- | --- | --- | --- |
| **MO2** | -0.043 | 0.005 | 0.000 |
| **MO3** | -0.398 | 0.039 | 0.000 |
| **SC2** | -0.060 | 0.016 | 0.000 |
| **SC3** | -0.230 | 0.020 | 0.000 |
| **UA2** | -0.044 | 0.005 | 0.000 |
| **UA3** | -0.171 | 0.018 | 0.000 |
| **PD2** | -0.069 | 0.008 | 0.000 |
| **PD3** | -0.344 | 0.058 | 0.000 |
| **AD2** | -0.057 | 0.018 | 0.001 |
| **AD3** | -0.187 | 0.016 | 0.000 |
| **Constant** | 0.943 | 0.029 | 0.000 |
| **Class 2** | **β** | **Std. Err** | **P value** |
| **MO2** | -0.090 | 0.020 | 0.000 |
| **MO3** | -0.428 | 0.045 | 0.000 |
| **SC2** | -0.105 | 0.023 | 0.000 |
| **SC3** | -0.262 | 0.020 | 0.000 |
| **UA2** | -0.063 | 0.022 | 0.003 |
| **UA3** | -0.186 | 0.022 | 0.000 |
| **PD2** | -0.084 | 0.011 | 0.000 |
| **PD3** | -0.298 | 0.037 | 0.000 |
| **AD2** | -0.048 | 0.022 | 0.028 |
| **AD3** | -0.202 | 0.038 | 0.000 |
| **Constant** | 0.743 | 0.079 | 0.000 |

**Table 5:** FMM - unweighted model.

**Table 6** Unweighted FMM, unweighted and weighted OLS (with application of population weights) to estimate the ‘pan-European’ value set for EQ-5D-3L

|  | **Unweighted model including interaction terms: N3, D1, I2, I3^2^** | | | **Weighted model including interaction terms: N3, D1, I2, I3^2^** | | |
| --- | --- | --- | --- | --- | --- | --- |
|  | **β** | **Std. Err** | **P value** | **β** | **Std. Err** | **P value** |
| **MO2** | -0.161 | 0.031 | 0.004 | -0.150 | 0.042 | 0.004 |
| **MO3** | -0.514 | 0.055 | 0.000 | -0.438 | 0.048 | 0.00 |
| **SC2** | -0.173 | 0.031 | 0.008 | -0.166 | 0.052 | 0.008 |
| **SC3** | -0.350 | 0.048 | 0.000 | -0.307 | 0.058 | 0.00 |
| **UA2** | -0.137 | 0.030 | 0.061 | -0.117 | 0.056 | 0.061 |
| **UA3** | -0.270 | 0.053 | 0.02 | -0.195 | 0.072 | 0.020 |
| **PD2** | -0.170 | 0.023 | 0.001 | -0.154 | 0.032 | 0.001 |
| **PD3** | -0.440 | 0.034 | 0.000 | -0.400 | 0.039 | 0.000 |
| **AD2** | -0.142 | 0.024 | 0.039 | -0.109 | 0.047 | 0.039 |
| **AD3** | -0.320 | 0.044 | 0.003 | -0.236 | 0.063 | 0.003 |
| **N3** | -0.097 | 0.040 | 0.093 | -0.133 | 0.072 | 0.093 |
| **D1** | 0.105 | 0.035 | 0.054 | 0.071 | 0.033 | 0.054 |
| **I2** | -0.022 | 0.020 | 0.374 | -0.007 | 0.007 | 0.374 |
| **I3^2^** | 0.004 | 0.003 | 0.331 | 0.001 | 0.001 | 0.331 |
| **Constant** | 1.000 | 0.000 | 0.00 | 1.000 | 0.000 | 0.000 |

*P >0.05; MO2 1 if mobility is level 2; 0 otherwise All models; MO3 1 if mobility is level 3; 0 otherwise All models; SC2 1 if self-care is level 2; 0 otherwise All models; SC3 1 if self-care is level 3; 0 otherwise All models; UA2 1 if usual activities is level 2; 0 otherwise All models; UA3 1 if usual activities is level 3; 0 otherwise All models; PD2 1 if pain/discomfort is level 2; 0 otherwise All models; PD3 1 if pain/discomfort is level 3; 0 otherwise All models; AD2 1 if anxiety/depression is level 2; 0 otherwise All models; AD3 1 if anxiety/depression is level 3; 0 otherwise All models; N3 1 if any dimension is level 3; 0 otherwise; D1 Number of movements away from full health beyond the first (the number of dimensions at level 2 or 3 beyond the first and replaces the constant); I2 Number of dimensions at level 2 beyond the first; I3^2^ The square of the number of dimensions at level 3 beyond the first.

**Figure 1:** Residual plot for the basic OLS model

**Table 7** Unweighted and weighted OLS (with application of population weights) to estimate the **“pan-EU”** value set for EQ-5D-3L with exclusion of the UK value set

|  | **OLS model (unweighted)** | | | **OLS model (applied population weights)** | | |
| --- | --- | --- | --- | --- | --- | --- |
|  | **β** | **Std. Err** | **P value** | **β** | **Std. Err** | **P value** |
| **MO2** | -0.075 | 0.015 | <0.001 | -0.085 | 0.018 | 0.001 |
| **MO3** | -0.427 | 0.036 | <0.001 | -0.392 | 0.023 | <0.001 |
| **SC2** | -0.085 | 0.016 | <0.001 | -0.101 | 0.024 | 0.002 |
| **SC3** | -0.257 | 0.021 | <0.001 | -0.261 | 0.017 | <0.001 |
| **UA2** | -0.052 | 0.012 | 0.002 | -0.052 | 0.026 | **0.075** |
| **UA3** | -0.181 | 0.019 | <0.001 | -0.149 | 0.031 | 0.001 |
| **PD2** | -0.080 | 0.006 | <0.001 | -0.088 | 0.012 | <0.001 |
| **PD3** | -0.340 | 0.023 | <0.001 | -0.351 | 0.031 | <0.001 |
| **AD2** | -0.054 | 0.012 | 0.001 | -0.043 | 0.179 | 0.035 |
| **AD3** | -0.222 | 0.025 | <0.001 | -0.187 | 0.024 | <0.001 |
| **Constant** | 0.859 | 0.031 | <0.001 | 0.864 | 0.028 | <0.001 |

*P >0.05; MO2 1 if mobility is level 2; 0 otherwise All models; MO3 1 if mobility is level 3; 0 otherwise All models; SC2 1 if self-care is level 2; 0 otherwise All models; SC3 1 if self-care is level 3; 0 otherwise All models; UA2 1 if usual activities is level 2; 0 otherwise All models; UA3 1 if usual activities is level 3; 0 otherwise All models; PD2 1 if pain/discomfort is level 2; 0 otherwise All models; PD3 1 if pain/discomfort is level 3; 0 otherwise All models; AD2 1 if anxiety/depression is level 2; 0 otherwise All models; AD3 1 if anxiety/depression is level 3; 0 otherwise All models
